# Comparative Genetic Analyses of Human Rhinovirus C (HRV-C) Complete Genome from Malaysia

**DOI:** 10.3389/fmicb.2016.00543

**Published:** 2016-04-29

**Authors:** Yam Sim Khaw, Yoke Fun Chan, Faizatul Lela Jafar, Norlijah Othman, Hui Yee Chee

**Affiliations:** ^1^Department of Medical Microbiology and Parasitology, Faculty of Medicine and Health Sciences, Universiti Putra MalaysiaSerdang, Malaysia; ^2^Department of Medical Microbiology, Faculty of Medicine, University of MalayaKuala Lumpur, Malaysia; ^3^Department of Paediatrics, Faculty of Medicine and Health Sciences, Universiti Putra MalaysiaSerdang, Malaysia

**Keywords:** Malaysia, complete genome, HRV-C, genomic feature, phylogenetic, negative selection

## Abstract

Human rhinovirus-C (HRV-C) has been implicated in more severe illnesses than HRV-A and HRV-B, however, the limited number of HRV-C complete genomes (complete 5′ and 3′ non-coding region and open reading frame sequences) has hindered the in-depth genetic study of this virus. This study aimed to sequence seven complete HRV-C genomes from Malaysia and compare their genetic characteristics with the 18 published HRV-Cs. Seven Malaysian HRV-C complete genomes were obtained with newly redesigned primers. The seven genomes were classified as HRV-C6, C12, C22, C23, C26, C42, and pat16 based on the VP4/VP2 and VP1 pairwise distance threshold classification. Five of the seven Malaysian isolates, namely, 3430-MY-10/C22, 8713-MY-10/C23, 8097-MY-11/C26, 1570-MY-10/C42, and 7383-MY-10/pat16 are the first newly sequenced complete HRV-C genomes. All seven Malaysian isolates genomes displayed nucleotide similarity of 63–81% among themselves and 63–96% with other HRV-Cs. Malaysian HRV-Cs had similar putative immunogenic sites, putative receptor utilization and potential antiviral sites as other HRV-Cs. The genomic features of Malaysian isolates were similar to those of other HRV-Cs. Negative selections were frequently detected in HRV-Cs complete coding sequences indicating that these sequences were under functional constraint. The present study showed that HRV-Cs from Malaysia have diverse genetic sequences but share conserved genomic features with other HRV-Cs. This genetic information could provide further aid in the understanding of HRV-C infection.

## Introduction

Human rhinovirus (HRV) has been recognized as one of the respiratory viruses associated with common cold in humans since the late 1950s. HRV infection is a significant burden that is frequently associated with morbidity and mortality among hospitalized pediatric patients not only in Malaysia, but also worldwide (Etemadi et al., 2013). HRV has more than 100 serotypes, which have been clustered into three groups: HRV-A, B, and C (McIntyre et al., 2013). To date, HRV-A consists of 80 serotypes, HRV-B consists of 32 types, and HRV-C consists of 55 types (Picornaviridae Study Group, 2014).

HRV-C, which was discovered in 2006, is a new species of HRV and has led to the reemergence of research interest in HRV (Lamson et al., 2006; Lau et al., 2007). The unique characteristics of HRV-C that distinguish it from HRV-A and HRV-B include the lack of usage of both major and minor receptors for host binding (Bochkov et al., 2011), potential resistance to pleconaril, a higher GC content, and a series of nucleotide deletions in the VP1 region, which yields a shorter complete genome (Lau et al., 2007).

A clearer picture of biological properties of HRV-A and HRV-B has been revealed through several studies that utilized a large number of HRV-A (*n* > 80) and HRV-B (*n* > 30) complete genome sequences in the Genbank (Palmenberg et al., 2009, 2010). Currently, there are only 18 HRV-C complete genomes (with complete 5′ non-coding region (NCR), 3′ NCR and open reading frame (ORF) sequences) available. This has greatly hampered the further study of this virus. Only partial and complete HRV-C VP1 and VP4/VP2 sequences have been largely published in the Genbank, hence, no in-depth analysis of the genomic features is possible.

Many studies have reported higher occurrence of HRV-C than HRV-A and HRV-B in severe respiratory diseases (McErlean et al., 2007; Miller et al., 2009). In addition, the association of specific HRV sequences with clinical severity is not well understood. To date, most of the published studies focused on the HRV-C capsid regions. Thus, a comparative sequence analysis using HRV-C complete genomes is an effective approach to gain more insight into this virus. In the present study, seven HRV-C complete genomes from children with respiratory tract infections in Malaysia were sequenced and comparative analyses of Malaysian isolates with other HRV-Cs were performed. This is the first report on the complete genome sequencing and comparative analyses of HRV-C from Malaysia. Seven HRV-C complete genome sequences were successfully amplified in the present study and comparative genetic analyses demonstrated that all HRV-Cs, as well as Malaysian isolates, share the same genomic features even though their nucleotide sequences are highly variable. This genetic information is useful for future studies of HRV-C pathogenesis and undoubtedly will make a significant contribution in the understanding of rhinovirus genomics.

## Materials and methods

### Patient recruitment and sample collection

Seven nasopharyngeal aspirate (NPA) samples were collected from children with respiratory tract infection at University Malaya Medical Center (UMMC) in Malaysia between October 2010 and May 2011 (Medical Ethic Approval no. 788.3) (Chan et al., 2012). These samples were previously confirmed as HRV-C based on the sequence of the VP4/VP2 region (Chan et al., 2012). RNA was extracted using QIAamp Viral RNA Mini Kit (Qiagen, Germany) according to the manufacturer's instructions.

### Redesigning primers

Three HRV-C complete genomes (accession number: EF582385, EF582386, and EF582387) were aligned by using ClustalW implemented in the MEGA6 program package (Tamura et al., 2013). Primers were modified and redesigned based on the primers used by Lau et al. (2007), and the redesigned primers were based on conserved regions to generate amplicon of less than 1 kb (Supplementary Table 1).

### Complete genome sequencing

cDNA was synthesized using the SuperScript III kit (Invitrogen, USA) according to the manufacturer's instructions with minor modifications. A combined random priming and oligo (dT) priming strategy was utilized to produce cDNA. The reaction mixture contained 2.75 μL of RNA, a mixture of 5 μM random hexamer (Thermo Scientific, USA) and 0.5 μM oligo-dT_18_ (Thermo Scientific, USA) (a ratio of 10:1), 0.5 mM dNTP, 1X first strand buffer (250 mM Tris-HCl pH 8.3, 375 mM KCl, 15 mM MgCl_2_), 5 mM DTT, 10 U RNaseOUT (Invitrogen, USA), 50 U SuperScript III reverse transcriptase, and nuclease-free water to a final volume of 5 uL. The mixture was incubated at 25°C for 10 min, followed by incubation at 55°C for 60 min, and a final incubation at 70°C for 15 min.

PCR amplification was performed with 1 μL cDNA, 1X Colorless GoTaq Flexi Buffer, 3 mM MgCl_2_, 0.2 mM dNTP (Fermentas, USA), 1.0 μM of each forward and reverse primer, and 1 U GoTaq Flexi DNA Polymerase (Promega, USA) for a final volume of 25 μL. This reaction was subjected to thermal cycling at 95°C for 5 min, followed by 35 cycles each consisting of denaturation at 95°C for 1 min, annealing for 1 min (annealing temperature varies with different primers in Supplementary Table 1), and extension at 72°C for 1 min and a final extension at 72°C for 5 min using MyCycler Thermal Cycler (BioRad, USA). The 5′ and 3′ NCRs of the viral genome were amplified using the 5′ rapid amplification of cDNA ends (RACE) system and 3′ RACE system (Invitrogen, USA), respectively, according to manufacturer's protocol.

The PCR products were gel-purified using the gel/PCR DNA fragments extraction kit (Geneaid, Taiwan). These products were bi-directionally sequenced with both forward and reverse primers by First Base Laboratories Sdn Bhd (Selangor, Malaysia). NCRs and the insufficient concentration of purified PCR products were cloned into the yT&A vector (Yeastern, Taiwan) and transformed into Top 10F' *Escherichia coli* following the manufacturer's protocol. Then, plasmids from three positive bacteria clones were extracted and purified using the High-Speed Plasmid Mini Kit (Geneaid, Taiwan). These positive plasmids were sent to First Base Laboratories Sdn Bhd (Selangor, Malaysia) for sequencing using ABI3770 sequencer (Applied Biosystems, USA).

### Genome alignment

The sequences were edited and assembled with the use of BioEdit version 7.1.11 software (Hall, 1999). Eighteen HRV-C complete genome references (with complete 5′ NCR, 3′ NCR and ORF) up until January 2016 were retrieved from the GenBank. These HRV-Cs were used in comparative analysis and are referred to as “other HRV-Cs.” Alignment of the seven HRV-C gene sequences completed in this study and the other HRV-Cs complete genomes was performed by using ClustalW implemented in the MEGA6 program package (Tamura et al., 2013).

### Phylogenetic analysis

JmodelTest 2.1.1 (Darriba et al., 2012) was used to determine the best-fit nucleotide substitution model. The maximum likelihood phylogenetic tree was constructed using General Time Reversible (GTR) as the best substitution model with gamma distributed and invariant sites. To construct the phylogenetic trees, bootstrap values calculated from 1000 trees were implemented in the MEGA6 program package (Tamura et al., 2013). The bootstrap values of 70 or higher were considered as significant clustering.

### Pairwise similarity

Pairwise similarity among the nucleotide of HRV-Cs complete genomes and complete coding sequences, and complete coding deduced amino acid sequences were calculated with the use of the GeneDoc 2.7.000 software (Nicholas et al., 1997).

### Sequence analyses

The nucleotide sequences of Malaysian isolates and other HRV-Cs were translated into deduced amino acid sequences using MEGA6 program package (Tamura et al., 2013). The deduced amino acid and NCR sequences of Malaysian isolates and other HRV-Cs were aligned with reference sequences: HRV-16 (accession number: L24917, HRV-A), HRV-2 (accession number: X02316, HRV-A), HRV-1 (accession number: FJ445111, HRV-A), HRV-1B (accession number: D00239, HRV-A), HRV-3 (accession number: EF173422, HRV-B), HRV-14 (accession number: NC_001490, HRV-B), HRV-70 (accession number: DQ473489, HRV-B), or coxsackievirus B3 (accession number: M33854, CV-B3) using ClustalW implemented in the MEGA6 program package (Tamura et al., 2013) to determine the functional motifs.

### Selective pressure analysis

The synonymous (dS) and non-synonymous (dN) changes at every codon of all the HRV-C complete coding sequences, including seven Malaysian isolates (*n* = 25), were estimated using three different selective pressure analyses implemented in DataMonkey (http://www.datamonkey.org) (Pond and Frost, 2005). The analyses were single likelihood ancestor counting (SLAC), fixed effects likelihood (FEL), and internal fixed effects likelihood (IFEL). These methods were conducted using the GTR model of nucleotide substitution and neighbor-joining method to determine the rate for dN and dS. In addition, the estimation of positive and negative selections was determined using *p*-value of 0.1. The synonymous rate exceeding the non-synonymous rate was considered as negative selection (dS > dN), while the positive selection was defined as when the non-synonymous rate exceeds the synonymous rate (dN > dS). Neutral selection was defined when the non-synonymous rate equals to the synonymous rate (dN = dS).

### Nucleotide sequence accession numbers

The complete genomes of seven Malaysian isolates were deposited in the GenBank under the accession numbers KF734978, KJ675505, KJ675506, KJ675507, KP890662, KP890663, and KP890664.

## Results

### Malaysian isolates showed highly diverse nucleotide sequences

Complete genomes of seven HRV-Cs (1515-MY-10, 1570-MY-10, 3430-MY-10, 3805-MY-10, 7383-MY-10, 8097-MY-11, and 8713-MY-10) were successfully sequenced in this study. Similar to other HRV-Cs, the nucleotide length of the Malaysian isolates complete genomes ranged from 7087 to 7127 bp, with an ORF that encoded for a polyprotein of 2139–2155 amino acids. Complete 5′ NCR and 3′ NCR lengths were 611–640 bp and 41–52 bp, respectively. The GC content of all the isolates was 42.3–43.8% (Supplementary Table 2).

Malaysian isolates complete genomes and complete coding sequences showed nucleotide similarity of 63–81% among themselves and 63–96% with other HRV-Cs (Table 1). In addition, Malaysian isolates demonstrated complete coding deduced amino acids similarity of 66–91% among themselves and 65–99% with other HRV-Cs. These results show that immense differences in both nucleotide and deduced amino acids sequences were observed among Malaysian isolates. Low nucleotide pairwise sequence similarity with different types of the other HRV-Cs suggests that Malaysian HRV-Cs are genetically diverse.

**Table 1:** **Nucleotide and deduced amino acid similarity between Malaysian isolates and HRV-C references**.

**HRV-C**	**Type assignment**	**Similarity (%)**																				
		**1515-MY-10**			**1570-MY-10**			**8713-MY-10**			**3430-MY-10**			**7383-MY-10**			**3805-MY-10**			**8097-MY-11**		
		**a**	**b**	**c**	**a**	**b**	**c**	**a**	**b**	**c**	**a**	**b**	**c**	**a**	**b**	**c**	**a**	**b**	**c**	**a**	**b**	**c**
1515-MY-10	C6	–	–	–	–	–	–	–	–	–	–	–	–	–	–	–	–	–	–	–	–	–
1570-MY-10	C42	64	64	66	–	–	–	–	–	–	–	–	–	–	–	–	–	–	–	–	–	–
8713-MY-10	C23	65	64	67	68	68	74	–	–	–	–	–	–	–	–	–	–	–	–	–	–	–
3430-MY-10	C22	75	75	85	64	64	66	64	64	67	–	–	–	–	–	–	–	–	–	–	–	–
7383-MY-10	pat16	81	81	91	64	64	66	64	64	67	75	75	85	–	–	–	–	–	–	–	–	–
3805-MY-10	C12	64	63	66	77	76	87	69	69	75	63	64	66	64	64	66	–	–	–	–	–	–
8097-MY-11	C26	63	64	66	64	64	68	66	66	70	64	65	67	63	64	66	64	64	68	–	–	–
KF958310/6331	C2	64	64	65	65	65	67	66	65	69	64	64	67	64	64	66	64	64	67	67	67	72
EF186077/QPM	C3	85	84	95	65	65	66	65	65	67	75	75	86	81	80	90	64	64	67	64	65	67
EF582385/024	C4	64	64	68	66	66	70	67	66	71	64	64	68	64	64	68	66	66	70	65	66	70
EF582386/025	C5	65	64	68	64	64	67	66	66	68	65	65	69	65	64	68	65	65	67	64	65	67
EF582387/026	C6	96	96	99	64	64	66	65	64	67	76	75	85	81	81	91	64	64	66	64	64	67
JF317016/LZ651	C6	94	94	98	64	64	66	64	64	67	75	75	85	81	80	91	64	64	66	64	64	66
DQ875932/NY-074	C7	77	77	87	63	63	65	64	64	67	77	75	85	77	77	87	64	64	66	63	64	66
GQ223227/N4	C8	64	65	67	71	71	78	68	68	74	64	64	67	64	64	67	71	71	78	64	65	68
GQ223228/N10	C9	64	64	66	64	65	67	65	65	69	63	63	66	63	64	66	64	64	68	71	71	78
EU840952/CL170085	C11	65	65	68	64	64	67	65	64	68	65	65	68	65	64	67	65	64	67	64	64	67
JF317017/LZY101	C12	63	64	66	77	77	87	69	69	75	64	64	66	64	64	66	92	92	98	64	65	68
JF317014/LZY79	C15	64	64	67	69	69	75	73	73	83	64	64	67	65	64	68	68	68	74	65	66	68
GU219984/W10	C15	64	64	68	69	69	75	73	73	83	64	64	68	65	65	68	68	68	75	65	66	68
JF317013/LZ269	C25	64	64	68	68	68	75	72	72	81	64	64	68	64	63	67	69	69	76	65	66	69
JN205461/WA823M02	C39	69	68	75	64	64	67	65	65	69	70	69	75	69	68	74	64	64	68	65	65	68
KF958311/2536	C41	65	65	69	68	68	74	74	74	83	65	65	68	65	64	68	68	69	75	66	66	69
JF317015/LZ508	C51	63	64	66	64	65	68	65	65	69	63	64	67	63	64	67	64	64	68	76	76	86
JX291115/JAL-1	C51	63	64	65	64	65	69	66	67	72	63	64	66	63	63	66	65	65	70	72	71	78

*^a^Complete genome nucleotide similarity*.

*^b^Complete coding nucleotide similarity*.

*^c^Complete coding deduced amino acid similarity*.

### Phylogenetic analysis based on all the available complete genomes showed a different clustering pattern compared to VP1 gene

**Figure 1:**
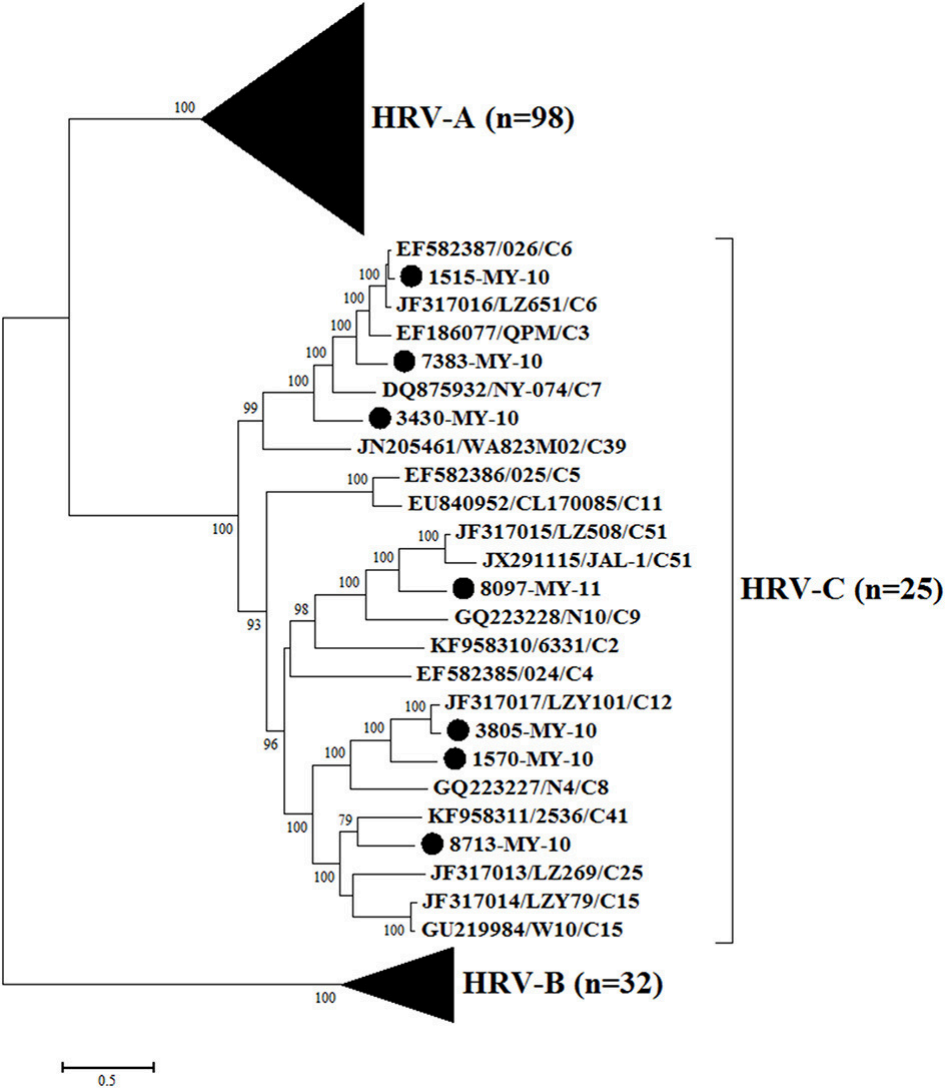
**Phylogenetic analysis of complete genome sequences of HRV-A, B, and C and seven Malaysian isolates used in this study**. The Malaysian isolates were denoted as (•). Tree was constructed by using maximum likelihood method and General Time Reversible as substitution model with gamma distributed with invariant sites rate, with 1000 bootstrap replicates implemented in the program MEGA version 6. Black triangles represent HRV-A and HRV-B isolates, respectively. Only bootstrap values with higher than 70% were shown.

Phylogenetic analysis of HRV-C complete genomes showed no clear geographical or temporal segregation of the Malaysian isolates and other HRV-Cs (Figure 1). The analysis revealed that 1515-MY-10 was closely related to 026 from Hong Kong, which has been classified as HRV-C6, and the nucleotide similarity was as high as 96% for the complete genome (Table 1). In addition, isolate 7383-MY-10 was also clustered with a group of C6 from Hong Kong and China and one HRV-C3 (QPM) from Australia. Isolate 3430-MY-10 showed a similar grouping pattern with 7383-MY-10, which this isolate grouped with an additional closer strain, NY-074 (C7) from USA compared to 7383-MY-10, while 8097-MY-11 grouped with C51 lineage from China and USA. Besides, isolate 3805-MY-10, which was closely clustered with LZY101 (C12) from China, also showed 92% of nucleotide similarity in the complete genome (Table 1). Isolate 1570-MY-10 was grouped together with 3805-MY-10 and LZY101 (C12), whereas 8713-MY-10 demonstrated a closer relationship with 2536 (C41) from the USA (Figure 1). Despite the obvious clustering pattern, the nucleotide similarities between 7383-MY-10, 3430-MY-10, 8097-MY-11, 1570-MY-10, 8713-MY-10 and their respective HRV-C in the clustering were only 74–81% (Table 1).

**Figure 2:**
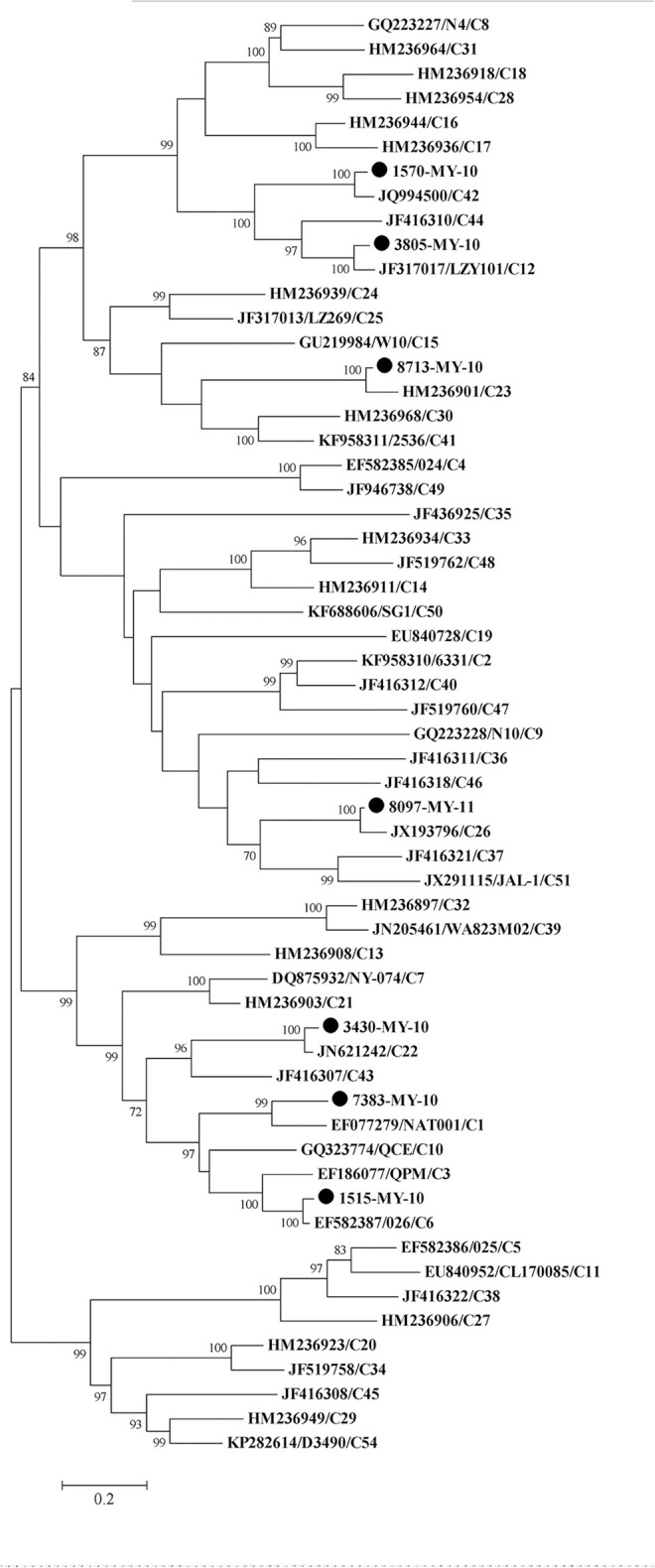
**Phylogenetic analysis of VP1 region of HRV-C and seven Malaysian isolates used in this study**. The Malaysian isolates were denoted as (•). Tree was constructed using maximum likelihood method and General Time Reversible as substitution model with gamma, distributed with invariant sites rate with 1000 bootstrap replicates, implemented in the program MEGA version 6. Only bootstrap values of higher than 70% were shown.

To perform the phylogenetic analysis based on the VP1 gene, a phylogenetic tree was built using the HRV-C complete VP1 gene from the available sequences in the Genbank. While there were many VP1 sequences in the Genbank for a single HRV-C type, only one VP1 sequence was chosen to represent each HRV-C type. Similar to the tree drawn with complete genome, 1515-MY-10 and 3805-MY-10 were still grouped with C6 and C12, respectively (Figures 1, 2); however, isolate 1570-MY-10 was later grouped with C42, 3430-MY-10 with C22, 7383-MY-10 with C1, 8097-MY-11 with C26, and 8713-MY-10 with C23 (Figure 2). The observed differences were due to the absence of C1, C22, C23, C26 and C42 complete genomes in Genbank.

### Pairwise similarity analysis revealed five new HRV-C complete genomes

Majority of the Malaysian isolates were consistent with pairwise distance threshold classification (Simmonds et al., 2010). They displayed more than 90% similarity with their respective HRV-C reference in both VP4/VP2 and VP1 nucleotide sequences, except for 7383-MY-10 (Supplementary Table 3). In a previous phylogenetic analysis, 7383-MY-10 demonstrated a closer relationship with pat16 in VP4/VP2 sequence (Chan et al., 2012). In this analysis, isolate 7383-MY-10 showed a highly similar result to pat16 with 93% in VP4/VP2 sequence, but did not show more than 90% similarity with any available HRV-C VP1 sequences at nucleotide level. The most similar HRV-C VP1 nucleotide sequence available in Genbank to 7383-MY-10 was HRV-C1 (NAT001, accession number: EF077279), for which the similarity was only 83%. Pat16 is one of the provisional HRV-Cs awaiting HRV-C type classification due to the absence of VP1 sequence (Picornaviridae Study Group, 2014).

Taken together the phylogeny and pairwise similarity analyses, the seven Malaysian isolates in this study, 1515-MY-10, 3805-MY-10, 3430-MY-10, 8713-MY-10, 8097-MY-11, 1570-MY-10, and 7383-MY-10 were classified as HRV-C6, C12, C22, C23, C26, C42, and pat16, respectively. Since only two Malaysian isolates, 1515-MY-10 and 3805-MY-10 showed high complete genome sequence similarity with their respective HRV-C references, 026/C6 (accession number: EF582387) and LZY101/C12 (accession number: JF317017), therefore, the remaining five HRV-C isolates from Malaysia are likely to represent newly sequenced HRV-C complete genomes.

### Malaysian isolates shared similar functional and genetic features with other HRV-Cs

As HRV-C is not readily propagated in typical culture system, the immunogenic sites of HRV-C were predicted using comparative sequence analysis of HRV-A (HRV-2, accession number: X02316) (Appleyard et al., 1990) and HRV-B (HRV-14, accession number: K02121) (Rossmann et al., 1985). Similar to other HRV-Cs, the Malaysian isolates were shorter in length as compared to HRV-A and HRV-B due to several deletions in the VP1 loops (Table 2). These deleted regions are BC, DE, and HI loops. BC and DE loops are known as immunogenicity determinants in HRV-A and HRV-B, while HI loop is not related to immunogenic site; thus, HI loop is excluded from Table 2. The locations of immunogenic sites of HRV-A are different from HRV-B, except for position 85. Five (positions 85, 91, 95, 138, and 139) of six HRV-B immunogenic sites were deleted in HRV-Cs, whereas only two (positions 85 and 86) of nine HRV-A immunogenic sites were deleted in the Malaysian isolates and other HRV-Cs VP1 protein.

**Table 2:** **Summary of predicted HRV-C deduced amino acids and their positions in the VP1 region based on HRV-A (Appleyard et al., 1990) and -B immunogenic sites (Rossmann et al., 1985)**.

**HRV**	**Position**^#^														
	**83**	**85^a^**	**86^a^**	**91^b^**	**92^a^**	**95^b^**	**138**	**139**	**264**	**267**	**268**	**269**	**276**	**278**	
HRV-A	–	T	L	–	E	–	–	–	K	D	R	S	T	P	
HRV-B	Q	K	–	D	–	E	D	S	–	–	–	–	–	–	
	Position^*^														
	**83**	**NP**^a^	**NP**^a^	**NP**^b^	**84**^a^	**NP**^b^	**NP**	**NP**	**248**	**258**	**259**	**260**	**267**		**269**
Other HRV-Cs	A/D/E/K/N/Q/R/S/T	^Ø^	^Ø^	^Ø^	D/E/N/Q/R/S	^Ø^	^Ø^	^Ø^	H/N	A/D/E/K/N/P/T/V	L/V	E/I/K/R/T	A/F/H/K/P/T/Y		A/D/E/G/N/P/Q/T
Malaysian isolates	D/K/N/P/R	^Ø^	^Ø^	^Ø^	E/N/Q/S/T	^Ø^	^Ø^	^Ø^	H/N	A/K/T/V	L	E/K/T	F/H/P/S/Y		D/E/N/R/T
Loop	–	BC	BC	BC	BC	BC	DE	DE	–	–	–	–	–		–

# *Position is according to reference sequence, HRV-A (HRV-2, accession number: X02316); HRV-B (HRV-14, accession number: K02121)*.

* *Position is according to reference sequence, HRV-C024 (accession number: EF582385)*.

a Denotes Nim-1A for HRV-A &

b *Denotes Nim-1A for HRV-B, otherwise denotes Nim-1B for HRV-A and -B*.

(Ø) *, deletion*.

Malaysian isolates shared six out of seven and three out of nine conserved deduced amino acids with HRV-A and HRV-B major receptor binding sites, respectively (Table 3). The only dissimilar deduced amino acid of Malaysian isolates with HRV-A major receptor binding sites was located at position 180 in VP3 region (according to HRV-A). Malaysian isolates displayed several different deduced amino acids such as alanine (A), lysine (K), or serine (S) at position 180 in VP3 region, whereas HRV-A has proline (P) at that position. The other HRV-Cs shared five out of seven deduced amino acids with HRV-A major receptor binding sites (Lau et al., 2007). Intriguingly, an additional deduced amino acid in the HRV-A major receptor binding site, threonine (T), was displayed by isolate 8713-MY-10/C23 in the position 179 of VP3 region (according to HRV-A). On the other hand, Lys_224_ (K) amino acid in VP1 region was not seen in Malaysian isolates. The absence of this amino acid was due to the deletion of HI loop in the VP1 protein (data not shown).

**Table 3:** **Comparison of potential deduced amino acids in the VP1 and carboxy-terminal VP3 regions involved in HRV-A and B major receptor binding with Malaysian isolates and other HRV-Cs**.

**HRV**	**VP3**							**VP1**				
	**Amino acid position**											
	**179**	**180**		**181**		**182**		**148**	**151**		**213**	
HRV-A major receptor	T^a^	P		D^b^		A/I/K/M/N^b^/S^b^/T		G^b^	A^b^/I/F/K/L/T^b^/V		A/D^b^/H	
	**178**	**179**		**180**		**181**		**133**	**136**		**200**	
Other HRV-Cs^*^	K/R	A/G/K/R/S		D^b^/S		D/E/K/N^b^/S^b^		G^b^	A^b^/G/H/P/S/T^b^/V		D^b^/G	
Malaysian isolates^*^	K/R/T^a^	A/K/S		D^b^		D/N^b^/S^b^		G^b^	A^b^/G/P/T^b^		D^b^	
	**Amino acid position**											
	**177**	**178**	**179**		**180**		**226**	**155**	**159**	**206**		**217**
HRV-B major receptor	D	K^b^/P	D^b^		T		Q/S	H/P^b^	K/N	H		V^c^
	**178**	**179**	**180**		**181**		**225**	**132**	**136**	**186**		**197**
Other HRV-Cs^*^	K/R	A/G/K^b^/R/S	D^b^/S		D/E/K/N/S		H/P	P^b^	A/G/H/P/S/T/V	A/I/K/L/N/R/S/T/V		A/E/L/P/Q/R/S/T/V^c^
Malaysian isolates^*^	K/R/T	A/K^b^/S	D^b^		D/N/S		P	P^b^	A/G/P/T	A/I/T/V		E/L/P/Q/S

*Major receptor binding positions of HRV-A and HRV-B were described by Kolatkar et al. (1999)*.

* *Position is according to reference sequence, HRV-C024 (accession number: EF582385)*.

a *Additional deduced amino acid similarity is shown by Malaysian isolate (8713-MY-10)*.

b *Similar deduced amino acids shared by HRV-A and B major receptor with Malaysian isolates and other HRV-Cs*.

c *Additional deduced amino acid displayed by other HRV-Cs but not in Malaysian isolates*.

Of the 25 amino acids, 15 and 14 amino acids corresponding to the pleconaril antiviral binding sites of HRV-A and HRV-B, respectively, were found in Malaysian isolates and other HRV-Cs (Table 4; Ledford et al., 2004). Also, these HRV-Cs demonstrated seven antiviral binding sites that were not analogous to HRV-A or HRV-B. These antiviral binding sites were located at positions 93, 113, 127, 166, 169, 221, and 243 (according to HRV-C024 accession number: EF582385). The putative antiviral binding sites of the Malaysian isolates and other HRV-Cs were superimposable onto the binding sites of HRV-A and HRV-B based on the same crucial C_α_ backbone present in each HRV group (Basta et al., 2014a). A minority group of HRV-Bs displaying Phe_152_ (F) and Leu_191_ (L) in the VP1 sequence were found to be associated with resistance to pleconaril (Ledford et al., 2004). Phe_152_ of HRV-B is equivalent to HRV-C Phe_129_. Four Malaysian isolates (1515-MY-10/C6, 3430-MY-10/C22, 8713-MY-10/C23, and 7383-MY-10/pat16) and the majority of other HRV-Cs displayed only Phe_129_ amino acid but not Leu_191_ in the VP1 sequence (data not shown).

**Table 4:** **Comparison of majority deduced amino acids involved in antiviral binding against pleconaril for HRV-A and HRV-B with Malaysian isolates and other HRV-Cs**.

**HRV**	**VP1 region**																								
	**Position**^#^																								
	**98**	**99**	**100**	**101**	**118**	**120**	**122**	**124**	**142**	**143**	**144**	**166**	**167**	**168**	**179**	**181**	**184**	**190**	**192**	**208**	**212**	**214**	**217**	**238**	**260**
HRV-A	I^a^	N^*a*^	L	Q^b^	F^a^	S	I^b^	L	Y	M^a^	Y^a^	A	S^a^	V^a^	F^a^	L	L	Y^a^	M^b^	T	N^a^	M^a^	L^b^	H	H
	Position^*^																								
	**104**	**105**	**106**	**107**	**124**	**126**	**128**	**130**	**150**	**151**	**152**	**174**	**175**	**176**	**186**	**188**	**191**	**197**	**199**	**215**	**219**	**221**	**224**	**245**	**267**
HRV-B	I^a^	N^a^	L	S	F^a^	S	Y	I^c^	A	M^a^	Y^a^	P^c^	S^a^	V^a^	F^a^	V	V	Y^a^	C	I^c^	N^a^	M^a^	M	H	G
	**Position**^@^																								
	**91**	**92**	**93**	**94**	**111**	**113**	**115**	**117**	**127**	**128**	**129**	**151**	**152**	**153**	**164**	**166**	**169**	**175**	**177**	**195**	**199**	**201**	**204**	**221**	**243**
Other HRV-Cs	I^a^	N^a^/S/T	F/Y	Q^b^	F^a^/Y	L/M	I^b^/V	I^c^/V	I/T/V	M^a^	F/Y^a^	P^c^	S^a^	I/V^a^	F^a^	I	M/T	Y^a^	I/M^b^/V	I^c^/T	D/N^a^	M^a^	L^b^	A/G/I/V	D/F/N/Y
Malaysian isolates	I^a^	N^a^/S	F	Q^b^	F^a^	L/M	I^b^/V	I^c^/V	I/T/V	M^a^	F/Y^a^	P^c^	S^a^	V^a^	F^a^	I	T	Y^a^	M^b^	I^c^	N^a^	M^a^	L^b^	A/I/V	F/Y

*These antiviral binding sites were described by Ledford et al. (2004)*.

a *Similar deduced amino acids shared in HRV-A and HRV-B with Malaysian isolates and other HRV-Cs*.

b *Similar deduced amino acids shared in HRV-A with Malaysian isolates and other HRV-Cs*.

c *Similar deduced amino acids shared in HRV-B with Malaysian isolates and other HRV-Cs*.

# *Position is according to reference sequence, HRV-16 (accession number: L24917)*.

* *Position is according to reference sequence, HRV-14 (accession number: K02121)*.

@ *Position is according to reference sequence, HRV-C024 (accession number: EF582385)*.

Many conserved motifs can be observed in the HRV genome. The genomic features of the Malaysian isolates and other HRV-Cs are summarized in Table 5. In the VP4 region, a conserved myristoylation motif, GAQVS motif, is essential for capsid protein assembly (Paul et al., 1987). This motif constitutes MGAQVS motif that is responsible for translation site of HRV polyprotein (Arden et al., 2010). Both of the motifs were identified in the Malaysian isolates and other HRV-Cs.

**Table 5:** **Summary of the genomic features of Malaysian isolates and other HRV-Cs**.

**Region**	**Conserved motif; Features**	**Characteristics**	
		**Malaysian isolates**	**Other HRV-Cs**
VP4	Myristylation site: GAQVS	Yes	Yes
	Translation site: MGAQVS	Yes	Yes
2A	Chymotrypsin-like protease with catalytic triad: H-D-C and GXCG motif	Yes	Yes
	Zinc ligand: C-C-C-H	Yes	Yes except N10/C9
2B	Two hydrophobic regions	Yes	Yes
2C	NTPase motif: GXXGXGKS	Yes	Yes
	Helicase: DDLXQ	One of the seven Malaysian isolates	Seven of 18 other HRV-Cs
	Cysteine rich motif (CX2-4CX6-8CX3-4C)	Yes	Yes
3A	Hydrophobic packing I-L-L-V-V	No	No
	Intermolecular salt bridge D-K-K	No	No
3B	Phosphodiester bond: Y_3_	Yes	Yes
3C	Chymotrypsin-like protease with catalytic triad: H-E-C and GQCG motif	Yes	Yes
	Substrate binding pocket: GXH	Four of the seven Malaysian isolates	Nine of 18 other HRV-Cs
	RNA binding motif: KFRDI	Yes	Yes
3D	KDELR motif	Yes	Yes
	GMPSG motif	Yes	Yes
	YGDD motif	Yes	Yes
	FLKR motif	Yes	Yes except JAL-1/C51
5′ NCR	Initial sequence, UU(A/G)AA(A/G)C(U/A)G	Yes	Yes
	Two pyrimidine rich segments	Yes	Yes
3′ NCR	Domain Y	Yes	Yes

Malaysian isolates are likely to carry a chymotrypsin-like cysteine 2A protease with a catalytic triad, histidine (H), aspartic acid (D), and cysteine (C) overlapped with the GDCG motif that facilitates initial cleavage at its capsid polyprotein. It also causes shutdown of the host cell protein synthesis (Hughes and Stanway, 2000). A zinc ligand, C-C-C-H, that helps to bind a zinc ion firmly for enzymatic activities was identified in the 2A protein of the Malaysian isolates (Petersen et al., 1999). Two hydrophobic regions of the 2B protein were identified in the Malaysian isolates. The first hydrophobic region is postulated to form a cationic amphipathic alpha helix, which is the major determinant for permeabilization of a host's plasma membrane (Agirre et al., 2002). Meanwhile, the second hydrophobic region may serve as a transmembrane domain to inhibit host protein secretion (van Kuppeveld et al., 1997). In addition, a NTPase motif, crucial for NTP binding and composed of the GXPGXGKS sequence (Gorbalenya et al., 1988), was found in the 2C protein of the Malaysian isolates. The DDLXQ motif is crucial for putative helicase function of 2C protein (de Souza Luna et al., 2008). This motif was found in one of the Malaysian isolates (8713-MY-10/C23). The majority of the Malaysian isolates displayed the DDVXQ motif, while two of the Malaysian isolates (1570-MY-10/C42 and 3805-MY-10/C12) showed DDIXQ motif. It is postulated that these three motifs (DDLXQ, DDVXQ and DDIXQ) play the same role as the amino acid properties of L, V, and I are similar. In addition, the Malaysian isolates 2C protein showed a cysteine-rich motif (CX2-4CX6-8CX3-4C) resembling a zinc finger, which is important in viral RNA replication (Pfister et al., 2000). Overall, the features of the Malaysian isolates 2A, 2B, and 2C proteins are similar to those of the other HRV-Cs.

Since there are no three-dimensional structures of HRV-C 3A protein, the characteristics of HRV-C 3A protein were postulated based on another picornavirus, coxsackievirus. Hydrophobic packing and intermolecular salt bridges are important in coxsackievirus replication (Wessels et al., 2006). Malaysian isolates 3A protein displayed I-L-L-S-V instead of I-L-L-V-V, suggesting the absence of hydrophobic packing. In addition, no intermolecular salt bridge was identified in the Malaysian isolates 3A protein. Tyrosine, an important amino acid in the 3B protein for linking covalently with viral RNA through a phosphodiester bond (Ambros and Baltimore, 1978), was conserved in the Malaysian isolates. Similar to the 2A protein, 3C protein of Malaysian isolates was probably a chymotrypsin-like cysteine protease with slightly different catalytic triads, histidine (H), glutamic acid (E), and cysteine (C) overlapped with GQCG motif (Matthews et al., 1994). Additionally, a substrate binding pocket, GXH (Matthews et al., 1994), was found in the 3C protein of four of the Malaysian isolates (1515-MY-10/C6, 3430-MY-10/C22, 7383-MY-10/pat16, and 8097-MY-11/C26). Moreover, the Malaysian isolates 3C protein showed a conserved RNA binding motif, KFRDI (Shih et al., 2004). For the 3D protein, four conserved motifs; KDELR, GMPSG, YGDD, and FLKR (Appleby et al., 2005) were identified in Malaysian isolates. The KDELR motif constitutes part of a loop that joins the finger and thumb domain, which forms the active site of the 3D polymerase (Appleby et al., 2005). The YGDD motif is crucial for nucleotidyl transfer reaction (Beese and Steitz, 1991). Overall, the characteristics of the Malaysian isolates 3A, 3B, 3C, and 3D proteins are similar to those of the other HRV-Cs.

A nine bp relatively conserved nucleotides sequence, UU(A/G)AA(A/G)C(U/A)G, was identified in the initial sequence of each Malaysian isolate 5′ NCR, where a VPg protein binds to the first U of the sequence (Palmenberg et al., 2009) and initiates its translation. In addition, two pyrimidine-rich segments were observed in the Malaysian isolates. The first segment is postulated as a determinant of HRV-C pathogenecity as this segment is equivalent to neurovirulence tropism of poliovirus (Palmenberg et al., 2009); whereas, the second segment represents the HRV ribosome entry region which is important for translation initiation (Borman and Jackson, 1992). Malaysian isolates were found to share the same 5′ NCR characteristics with other HRV-Cs. Similar to other HRV-Cs, Malaysian isolates demonstrated domain Y in 3′ NCR (Pilipenko et al., 1992).

### HRV-C complete coding sequences were predominated by purifying selective pressure

**Figure 3:**
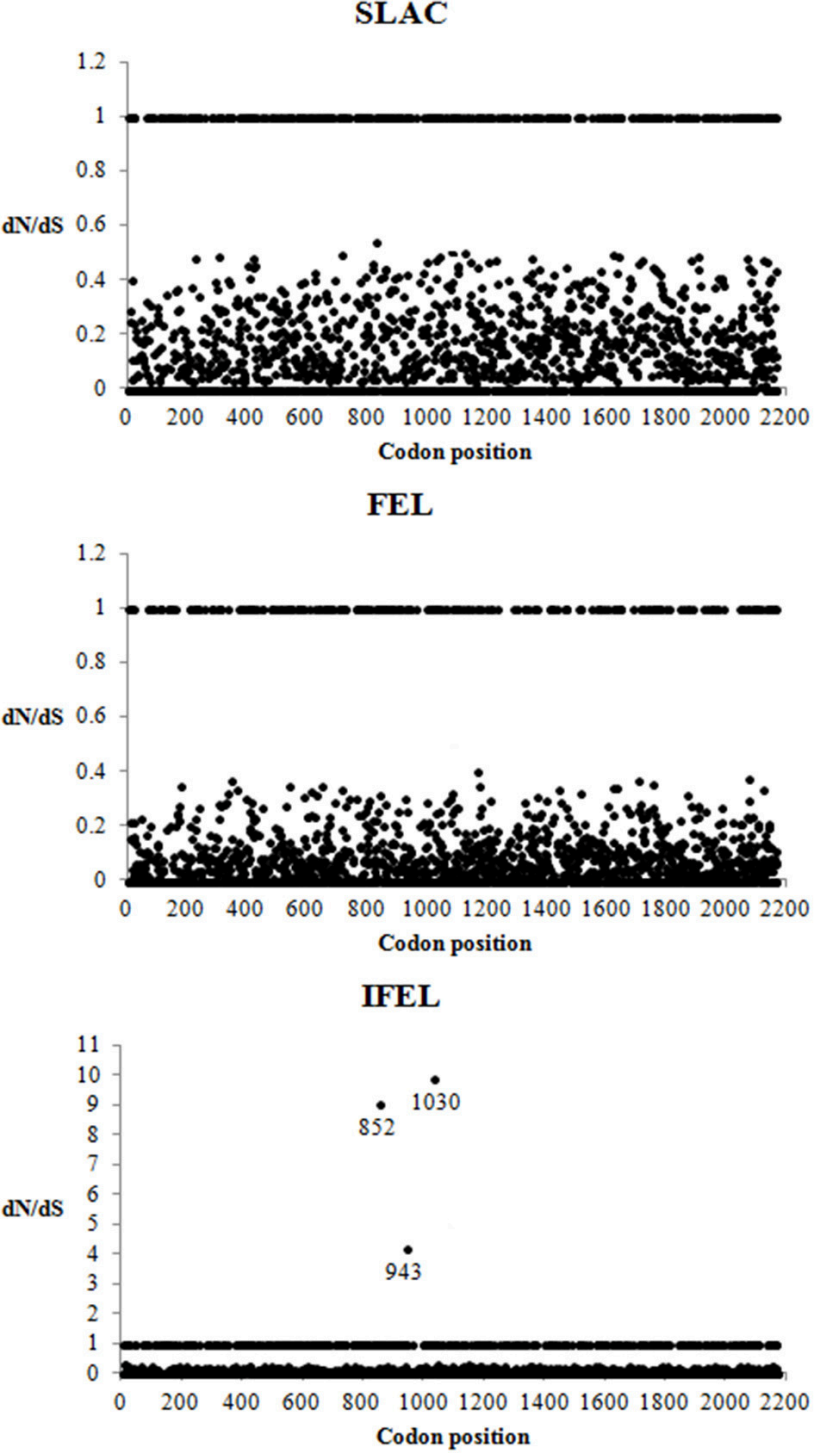
**Detection of selection pressures on complete coding sequences of HRV-C using SLAC, FEL, and REL methods**. dN/dS > 1, dN/dS = 1, and dN/dS < 1 indicated codons under positive, neutral and negative selection, respectively. The codons with positive selection were labeled with their codon position.

A total of 2168 codon sites of the Malaysian isolates and other HRV-Cs aligned complete coding sequence were utilized for the selective pressure analysis. Negative selection was found to be the dominant selective pressure on HRV-C complete coding sequences using SLAC, FEL, and IFEL methods at *P* < 0.1 level. These analyses (SLAC, FEL, and IFEL) found that 81% (1758/2168), 87% (1886/2168), and 79% (1716/2168) codon sites, respectively, were highly significant for purifying selections. The IFEL method demonstrated three positive selected sites but SLAC and FEL methods did not demonstrate any. These three positive selected sites were located in 852, 943, and 1030 codon position (Figure 3).

## Discussion

Respiratory infection is a significant burden in Malaysia (Khor et al., 2012). HRV infection, although usually associated with mild symptoms, can be associated with more severe acute respiratory infections and complications including asthma exacerbations (Lau et al., 2007). Due to the higher incidence of HRV-C in causing severe diseases and insufficient availability of HRV-C complete genome sequences for further analyses, the present study aimed to sequence the complete genome of Malaysian HRV-Cs and to perform genetic analyses of these viruses. These samples were previously confirmed as HRV-C based on the phylogenetic analysis of the VP4/VP2 region and designated as 1515-MY-10, 1570-MY-10, 3430-MY-10, 3805-MY-10, 7383-MY-10, 8097-MY-11, and 8713-MY-10 (Chan et al., 2012). Seven HRV-C complete genome sequences consisting of 5′ and 3′ NCR, and the ORF were successfully amplified in this study. These seven Malaysian isolates were isolated from patients displaying a range of respiratory diseases such as bronchiolitis, staphylococcal pneumonia and acute exacerbations of bronchial asthma (AEBA). Low nucleotide similarity was observed in Malaysian isolates when compared to other HRV-Cs suggesting that these isolates are genetically diverse. Similar genetic diversity has been reported in HRV-C as well as HRV-A and HRV-B (Palmenberg et al., 2009). In addition, the previous primer sets from Lau et al. (2007) were unable to amplify all of the Malaysian isolates, further demonstrating the diverse nucleotide sequences of Malaysian isolates.

Groupings in phylogenetic analysis of HRV-C complete genome were different from previous VP4/VP2 phylogenetic analysis (Chan et al., 2012) which clustered 1515-MY-10, 3805-MY-10, 3430-MY-10, 8713-MY-10, 8097-MY-11, 1570-MY-10, and 7383-MY-10 with HRV-C6, C12, C22, C23, C26, C42, and pat16, respectively. In the present study, phylogenetic analysis of VP1 gene demonstrated a similar clustering pattern with VP4/VP2 gene phylogenetic analysis, except for 7383-MY-10. 7383-MY-10 was clustered with C1 in the VP1 phylogenetic tree, and the similarity was only 83%. The similarity is inconsistent with the classification proposed by Simmonds et al. (2010) suggesting a paucity of representative VP1 sequences for pat16. On the other hand, several short VP1 and VP4/VP2 sequences of C22, C23, C26, and C42 are available in Genbank. These sequences showed at least 87% similarity with their respective Malaysian isolate sequences (1570-MY-10, 3430-MY-10, 8097-MY-11, and 8713-MY-10).

McErlean et al. (2007) determined that the length of HRV-C is shorter than HRV-A and HRV-B, as a result of several deletions in the BC, DE, and HI loops of VP1 region. Malaysian isolates have the same genome length as other HRV-Cs. The BC and DE loops are vital in major neutralization sites, especially Nim-1A and Nim-1B sites (Lau et al., 2007). The deletions of the putative neutralization sites in HRV-C remove the exposed loops and prevent the contact with host antibody; thus, HRV-C may have the possibility to escape host antibody recognition. This could be the reason why HRV-C is responsible for more severe respiratory diseases as compared to other HRVs (Basta et al., 2014b). Besides that, more deletions in HRV-C of HRV-B immunogenic sites were observed as compared with HRV-A sites, suggesting that a vaccine or antiviral targeting the neutralization sites for HRV-C could be designed based on HRV-A instead of HRV-B.

As for host receptor binding, Lau et al. (2007) reported that HRV-Cs shared five out of the seven and four out of the nine conserved residues with major receptors of HRV-A and HRV-B, respectively, based on a study by Kolatkar et al. (1999). The authors speculated that these HRV-Cs may not exploit the major receptor for host binding. One of the Malaysian isolates, 8713-MY-10/C23, showed that its putative major receptor binding sites were very similar to HRV-A major receptor binding sites, as compared to other HRV-Cs. We speculate that this Malaysian isolate may not utilize the HRV-A major receptor because the binding sites required three fully conserved amino acids (Thr_179_, Pro_180_, and Asp_181_) in the VP3 region (Laine et al., 2006), and this was not conserved in this isolate. Lack of amino acid similarity between Malaysian isolates and HRV-B major receptor binding site also suggests that the Malaysian isolates may not exploit this receptor either. In addition, the deletion of the HI loop of VP1 gene in the Malaysian isolates has removed the Lys_224_ amino acid, which is crucial for minor receptor binding (Lau et al., 2007). Bochkov et al. (2011) demonstrated that HRV-C was still able to attach to the target cells although ICAM-1 (major receptor) and LDLR (minor receptor) antibodies were used to block these receptors. Taken together, these observations may imply that major and minor receptors are not utilized by HRV-C for attachment. Overall, the Malaysian isolates, like other HRV-Cs, are unlikely to utilize both the major and minor receptors for host binding based on genome sequence analysis. Recently, cadherin-related family member 3, a transmembrane protein with unknown function, was discovered as a possible receptor for HRV-C (Bochkov et al., 2015).

Arden et al. (2010) demonstrated 11 and 12 residue differences of HRV-C (HRVC-QCE) compared with HRV-A and HRV-B, respectively, among the 25 important contact residues of the antiviral binding pocket. With the increasing number of HRV-Cs, the Malaysian isolates and other HRV-Cs demonstrated a higher number of similar residues with HRV-A and HRV-B with respect to the antiviral binding pocket. Basta et al. (2014a) showed that four unique antiviral residues, Phe/Tyr_96_, Leu/Met_116_, Ile/Val_130_, and Ile_169_ were only found in HRV-C, but not HRV-A and HRV-B. These residues were seen in the Malaysian isolates and are equivalent to Phe/Tyr_93_, Leu/Met_113_, Ile/Thr/Val_127_, and Ile_166_, respectively, in this study. Phe_96_ and Met_116_ of C15 have been shown to cause occasional steric clashes with some antiviral drugs (Basta et al., 2014a). According to the antiviral susceptibility pattern constructed by Basta et al. (2014a), Malaysian isolates and other HRV-Cs showed an additional unique residue, Ala/Gly/Ile/Val_221_ in their sequences compared with HRV-A and HRV-B. These different residues of HRV-C undoubtedly changed the characteristic of HRV-C VP1 protein structure for antiviral binding, as compared to HRV-A and HRV-B. In an antiviral study, Ledford et al. (2004) demonstrated that apparent resistance to pleconaril was developed from HRV-B with Phe_152_ and Leu_191_ amino acids in the VP1 region. In the following year, Ledford et al. (2005) concluded that the resistance effect was most profound if both Phe_152_ and Leu_191_ were present concurrently in HRV. In the present study, none of the Malaysian isolates and other HRV-Cs demonstrated the simultaneous presence of these two amino acids. Recently, Hao et al. (2012) showed that HRV-C15 with only Phe_129_ amino acid (same as HRV-B Phe_152_ amino acid) alone was capable of confering noticeable resistance to pleconaril. Most of the Malaysian isolates and other HRV-Cs demonstrated the presence of Phe_129_ amino acid in the VP1 sequence, suggesting that they are likely resistant to pleconaril.

Negative selection was the dominant selective pressure acting on the HRV-C complete coding sequences. This result is in agreement with Kistler et al. (2007), which reported that most of the HRV coding regions were under strong negative selective pressure. Besides that, most of the motifs in the complete coding sequences displayed low ratio of the number of non-synonymous substitutions per non-synonymous site to the number of synonymous substitutions per synonymous site. This indicates that these motifs have been under functional constraint throughout evolution. No positive selected sites were identified in HRV-C capsid region (Kuroda et al., 2015), whereas a positive selected site was reported in VP1 region of all HRVs complete coding sequences (86 HRV-A, 33 HRV-B, and 14 HRV-C) (Waman et al., 2014). A larger HRV-C dataset (*n* = 25) was exploited in the present study and several positive selective sites were identified using the IFEL method. One of these positive selected sites (852 codon position) was located in C-terminus of VP1 region, and it correlates with Waman et al. (2014). Another two positive selective pressures were found in 2A and 2B gene, respectively. The biological relevance of these three positive selected sites is hypothesized to increase in the HRV-C survival rate. For example, the positive selective site in C-terminus VP1 region is located on the outer surface, corresponding to the HRV-A antigenic site (269 position in HRV-C VP1 gene), and possibly interacts with host immune system (Table 2) resulting in the alteration of HRV-C immunogenicity. However, this hypothesis will require further support and verification from experimental data. For instance, the positively selected site is mapped within the Nim sites of HRV-A, it remains obscure whether this positive selective pressure affects the immunogenicity of HRV-C as the immunogenic determinants of HRV-C have not been identified. To our knowledge, no studies focusing on these selected sites in HRV-C have been reported. Future studies are necessary to determine the biological importance of these positive selected sites. In turn, these findings can identify the pathogenic role of HRV-C in causing severe diseases.

## Conclusion

Complete genomes of seven HRV-C Malaysian isolates have been sequenced and were classified as C6, C12, C22, C23, C26, C42, and pat16 based on the pairwise distance threshold classification. This is the first report of complete genomes of C22, C23, C26, C42, and pat16. This study found that the putative biological characteristics of the Malaysian isolates were similar to those of other HRV-Cs. Moreover, negative selective pressure was the predominant pressure acting on complete coding sequences of HRV-C, and this pressure conserved the functional motif of these HRV-Cs, although the nucleotides were diverse. These indicate that a similar treatment and control of HRV-C can be applied to patients infected with these wide ranges of HRV-Cs. This study has represented the most up-to-date information about HRV-C genomics and shown that although HRV-Cs have diverse genetic sequences, they share conserved genomic features. Further *in vitro* and *in vivo* study will be required to clarify the HRV-C antiviral resistance and receptor binding sites that were predicted based on the genome sequences.

## Author contributions

HYC, YFC, and NO participated in design of the study. YSK and FLJ performed the experiments. YSK, HYC, and YFC analyzed the data and drafted the manuscript. All authors revised and approved the final manuscript.

### Conflict of interest statement

The authors declare that the research was conducted in the absence of any commercial or financial relationships that could be construed as a potential conflict of interest.

## References

[B1] Agirre A. Barco A. Carrasco L. Nieva J. L. (2002). Viroporin-mediated membrane permeabilization. Pore formation by nonstructural poliovirus 2B protein. J. Biol. Chem. 277, 40434–40441. 10.1074/jbc.M205393200 12183456

[B2] Ambros V. Baltimore D. (1978). Protein is linked to the 5′end of poliovirus RNA by a phosphodiester linkage to tyrosine. J. Biol. Chem. 253, 5263–5266. 209034

[B3] Appleby T. C. Luecke H. Shim J. H. Wu J. Z. Cheney W. I. Zhong W. D.. (2005). Crystal structure of complete rhinovirus RNA polymerase suggests front loading of protein primer. J. Virol. 79, 277–288. 10.1128/JVI.79.1.277-288.2005 15596823 PMC538717

[B4] Appleyard G. Russell S. M. Clarke B. E. Speller S. A. Trowbridge M. Vadolas J. (1990). Neutralization epitopes of human rhinovirus type 2. J. Gen. Virol. 71, 1275–1282. 10.1099/0022-1317-71-6-1275 1693662

[B5] Arden K. E. Faux C. E. O'Neill N. T. McErlean P. Nitsche A. Lambert S. B.. (2010). Molecular characterization and distinguishing features of a novel human rhinovirus (HRV) C, HRVC-QCE, detected in children with fever, cough and wheeze during 2003. J. Clin. Virol. 47, 219–233. 10.1016/j.jcv.2010.01.001 20106717 PMC7108254

[B6] Basta H. A. Ashraf S. Sgro J. Y. Bochkov Y. A. Gern J. E. Palmenberg A. C. (2014a). Modeling of the human rhinovirus C capsid suggests possible causes for antiviral drug resistance. Virology 448, 82–90. 10.1016/j.virol.2013.10.004 24314639 PMC3878079

[B7] Basta H. A. Sgro J. Y. Palmenberg A. C. (2014b). Modeling of the human rhinovirus C capsid suggests a novel topography with insights on receptor preference and immunogenicity. Virology 448, 176–184. 10.1016/j.virol.2013.10.006 24314648 PMC3857591

[B8] Beese L. S. Steitz T. A. (1991). Structural basis for the 3′-5′exonuclease activity of Escherichia coli DNA polymerase I: a two metal ion mechanism. EMBO J. 10, 25–33. 1989886 10.1002/j.1460-2075.1991.tb07917.x PMC452607

[B9] Bochkov Y. A. Palmenberg A. C. Lee W. M. Rathe J. A. Amineva S. P. Sun X.. (2011). Molecular modeling, organ culture and reverse genetics for a newly identified human rhinovirus C. Nat. Med. 17, 627–633. 10.1038/nm.2358 21483405 PMC3089712

[B10] Bochkov Y. A. Watters K. Ashraf S. Griggs T. F. Devries M. K. Jackson D. J.. (2015). Cadherin-related family member 3, a childhood asthma susceptibility gene product, mediates rhinovirus C binding and replication. Proc. Natl. Acad. Sci. U.S.A. 112, 5485–5490. 10.1073/pnas.1421178112 25848009 PMC4418890

[B11] Borman A. Jackson R. J. (1992). Initiation of translation of human rhinovirus RNA: mapping the internal ribosome entry site. Virology 188, 685–696. 10.1016/0042-6822(92)90523-R 1316679

[B12] Chan Y. F. Jafar F. L. Nathan A. M. de Bruyne J. A. Hassan A. Nor'e S. S.. (2012). Diverse human rhinoviruses A and C from children with respiratory infections in Kuala Lumpur, Malaysia. J. Infection 64, 633–636. 10.1016/j.jinf.2012.03.011 22425558

[B13] Darriba D. Taboada G. L. Doallo R. Posada D. (2012). jModelTest 2: more models, new heuristics and parallel computing. Nat. Methods 9:772. 10.1038/nmeth.2109 22847109 PMC4594756

[B14] de Souza Luna L. K. Baumgarte S. Grywna K. Panning M. Drexler J. F. Drosten C. (2008). Identification of a contemporary human parechovirus type 1 by VIDISCA and characterization of its full genome. Virol. J. 5:26. 10.1186/1743-422X-5-26 18269761 PMC2270820

[B15] Etemadi M. R. Othman N. Savolainen-Kopra C. Sekawi Z. Wahab N. Sann L. M. (2013). Biodiversity and clinico-demographic characteristics of human rhinoviruses from hospitalized children with acute lower respiratory tract infections in Malaysia. J. Clin. Virol. 58, 671–677. 10.1016/j.jcv.2013.05.017 23932333 PMC7172529

[B16] Gorbalenya A. E. Koonin E. V. Donchenko A. P. Blinov V. M. (1988). A conserved NTP-motif in putative helicases. Nature 333:22. 10.1038/333022a0 2834648

[B17] Hall T. A. (1999). BioEdit, a user-friendly biological sequence alignment editor and analysis program Windows 95/98/NT. Nucleic Acid Symp. Ser. 41, 95–98. 10.1021/bk-1999-0734.ch008

[B18] Hao W. Bernard K. Patel N. Ulbrandt N. Feng H. Svabek C.. (2012). Infection and propagation of human rhinovirus C in human airway epithelial cells. J. Virol. 86, 13524–13532. 10.1128/JVI.02094-12 23035218 PMC3503113

[B19] Hughes P. J. Stanway G. (2000). The 2A proteins of three diverse picornaviruses are related to each other and to the H-rev107 family of proteins involved in the control of cell proliferation. J. Gen. Virol. 81, 201–207. 10.1099/0022-1317-81-1-201 10640559

[B20] Khor C. S. Sam I. C. Hooi P. S. Quek K. F. Chan Y. F. (2012). Epidemiology and seasonality of respiratory viral infections in hospitalized children in Kuala Lumpur, Malaysia: a retrospective study of 27 years. BMC Pediatr. 12:32. 10.1186/1471-2431-12-32 22429933 PMC3337250

[B21] Kistler A. L. Webster D. R. Rouskin S. Magrini V. Credle J. J. Schnurr D. P.. (2007). Genome-wide diversity and selective pressure in the human rhinovirus. Virol. J. 4:40. 10.1186/1743-422X-4-40 17477878 PMC1892812

[B22] Kolatkar P. R. Bella J. Olson N. H. Bator C. M. Baker T. S. Rossmann M. G. (1999). Structural studies of two rhinovirus serotypes complexed with fragments of their cellular receptor. EMBO J. 18, 6249–6259. 10.1093/emboj/18.22.6249 10562537 PMC1171688

[B23] Kuroda M. Niwa S. Sekizuka T. Tsukagoshi H. Yokoyama M. Ryo A.. (2015). Molecular evolution of the VP1, VP2, VP3 genes in human rhinovirus species C. Sci. Rep. 5:8185. 10.1038/srep08185 25640899 PMC4313092

[B24] Laine P. Blomqvist S. Savolainen C. Andries K. Hovi T. (2006). Alignment of capsid protein VP1 sequences of all human rhinovirus prototype strains, conserved motifs and functional domains. J. Gen. Virol. 87, 129–138. 10.1099/vir.0.81137-0 16361425

[B25] Lamson D. Renwick N. Kapoor V. Liu Z. Palacios G. Ju J.. (2006). MassTag polymerase-chain-reaction detection of respiratory pathogens, including a new rhinovirus genotype, that caused influenza-like illness in New York State during 2004-2005. J. Infect. Dis. 194, 1398–1402. 10.1086/508551 17054069 PMC7110122

[B26] Lau S. K. Yip C. C. Tsoi H. W. Lee R. A. So L. Y. Lau Y. L.. (2007). Clinical features and complete genome characterization of a distinct human rhinovirus (HRV) genetic cluster, probably representing a previously undetected HRV species, HRV-C, associated with acute respiratory illness in children. J. Clin. Virol. 45, 3655–3664. 10.1128/JCM.01254-07 17804649 PMC2168475

[B27] Ledford R. M. Collett M. S. Pevear D. C. (2005). Insights into the genetic basis for natural phenotypic resistance of human rhinoviruses to pleconaril. Antiviral Res. 68, 135–138. 10.1016/j.antiviral.2005.08.003 16199099

[B28] Ledford R. M. Patel N. R. Demenczuk T. M. Watanyar A. Herbertz T. Collett M. S.. (2004). VP1 sequencing of all human rhinovirus serotypes: insights into genus phylogeny and susceptibility to antiviral capsid-binding compounds. J. Virol. 78, 3663–3674. 10.1128/JVI.78.7.3663-3674.2004 15016887 PMC371056

[B29] Matthews D. A. Smith W. W. Ferre R. A. Condon B. Budahazi G. Slsson W.. (1994). Structure of human rhinovirus 3C protease reveals a trypsin-like polypeptide fold, RNA-binding site, and means for cleaving precursor polyprotein. Cell 77, 761–771. 10.1016/0092-8674(94)90059-0 7515772

[B30] McErlean P. Shackelton L. A. Lambert S. B. Nissen M. D. Sloots T. P. Mackay I. M. (2007). Characterisation of a newly identified human rhinovirus, HRV-QPM, discovered in infants with bronchiolitis. J. Clin. Virol. 39, 67–75. 10.1016/j.jcv.2007.03.012 17482871 PMC7172271

[B31] McIntyre C. L. Knowles N. J. Simmonds P. (2013). Proposals for the classification of human rhinovirus species A, B and C into genotypically assigned types. J. Gen. Virol. 94, 1791–1806. 10.1099/vir.0.053686-0 23677786 PMC3749525

[B32] Miller E. K. Khuri-Bulos N. Williams J. V. Shehabi A. A. Faouri S. Al Jundi I.. (2009). Human rhinovirus C associated with wheezing in hospitalised children in the Middle East. J. Clin. Virol. 46, 85–89. 10.1016/j.jcv.2009.06.007 19581125 PMC2759319

[B33] Nicholas K. B. Nicholas H. B. J. Deerfield D. W. (1997). GeneDoc: analysis and visualization of genetic variation, EMBNEW. NEWS 4, 14.

[B34] Palmenberg A. C. Rathe J. A. Liggett S. B. (2010). Analysis of the complete genome sequences of human rhinovirus. J. Allergy Clin. Immunol. 125, 1190–1199. 10.1016/j.jaci.2010.04.010 20471068 PMC2893015

[B35] Palmenberg A. C. Spiro D. Kuzmickas R. Wang S. Djikeng A. Rathe J. A.. (2009). Sequencing and analyses of all known Human Rhinovirus genomes reveal structure and evolution. Science 324, 55–59. 10.1126/science.1165557 19213880 PMC3923423

[B36] Paul A. V. Schultz A. Pincus S. E. Oroszlan S. Wimmer E. (1987). Capsid protein VP4 of poliovirus is N-myristoylated. Proc. Natl. Acad. Sci. U.S.A. 84, 7827–7831. 2825164 10.1073/pnas.84.22.7827 PMC299409

[B37] Petersen J. F. W. Cherney M. M. Liebig H. D. Skern T. Kuechler E. James M. N. G. (1999). The structure of the 2A proteinase from a common cold virus, a proteinase responsible for the shut-off of host-cell protein synthesis. EMBO J. 18, 5463–5475. 10.1093/emboj/18.20.5463 10523291 PMC1171615

[B38] Pfister T. Jones K. W. Wimmer E. (2000). A Cysteine-Rich motif in poliovirus protein 2C ATPASE is involved in RNA replication and binds zinc *in vitro*. J. Virol. 74, 334–343. 10.1128/JVI.74.1.334-343.2000 10590122 PMC111544

[B39] Picornaviridae Study Group (2014). Rhinovirus C. Available online at: http://www.picornastudygroup.com/types/enterovirus/hrv-c.htm.

[B40] Pilipenko E. V. Maslova S. V. Sinyakov A. N. Agol V. I. (1992). Towards identification of cis-acting elements involved in the replication of enterovirus and rhinovirus RNAs: a proposal for the existence of tRNA-like terminal structures. Nucleic Acids Res. 20:1739. 10.1093/nar/20.7.1739 1315956 PMC312265

[B41] Pond S. L. Frost S. D. (2005). Datamonkey, rapid detection of selective pressure on individual sites of codon alignments. Bioinformatics 21, 2531–2533. 10.1093/bioinformatics/bti320 15713735

[B42] Rossmann M. G. Arnold E. Erickson J. W. Frankenberger E. A. Griffith J. P. Hecht H. J.. (1985). Structure of a human common cold virus and functional relationship to other picornaviruses. Nature 317, 145–153. 10.1038/317145a0 2993920

[B43] Shih S. R. Chiang C. Chen T. C. Wu C. N. Hsu J. T. Lee J. C.. (2004). Mutations at KFRDI and VGK domains of enterovirus 71 3C protease affects its RNA binding and proteolytic activities. J. Biomed. Sci. 11, 239–248. 10.1007/BF02256567 14966374

[B44] Simmonds P. McIntyre C. Savolainen-Kopra C. Tapparel C. Mackay I. M. Hovi T. (2010). Proposals for the classification of human rhinovirus species C into genotypically assigned types. J. Gen. Virol. 91, 2409–2419. 10.1099/vir.0.023994-0 20610666

[B45] Tamura K. Stecher G. Peterson D. Filipski A. Kumar S. (2013). MEGA6, molecular evolutionary genetics analysis version 6.0. Mol. Biol. Evol. 30, 2725–2729. 10.1093/molbev/mst197 24132122 PMC3840312

[B46] van Kuppeveld F. J. M. Hoenderop J. G. J. Smeets R. L. L. Willems P. H. G. M. Dijkman H. B. P. M. Galama J. M. D.. (1997). Coxsackievirus protein 2B modifies endoplasmic reticulum membrane and plasma membrane permeability and facilitates virus release. EMBO J. 16, 3519–3532. 10.1093/emboj/16.12.3519 9218794 PMC1169977

[B47] Waman V. P. Kolekar P. S. Kale M. M. Kulkarni-Kale U. (2014). Population structure and evolution of rhinovirus. PLoS ONE 9:e88981. 10.1371/journal.pone.0088981 24586469 PMC3929619

[B48] Wessels E. Notebaart R. A. Duijsings D. Lanke K. Vergeer B. Melchers W. J. G.. (2006). Structure-function analysis of the coxsackievirus protein 3A identification of residues important for dimerization, viral RNA replication and transport inhibition. J. Biol. Chem. 281, 28232–28243. 10.1074/jbc.M601122200 16867984

