# Supplementary material for: Comparative Genetic Analyses of Human Rhinovirus C (HRV-C) Complete Genome from Malaysia
Source: Front Microbiol. 2016 Apr 29;7:543. doi: 10.3389/fmicb.2016.00543 (PMC4851184; doi:10.3389/fmicb.2016.00543)
Supplement: Supplementary file 2 [file Table2.DOC]

**Supplement Table 2. Genomic information of Malaysian isolates and other HRV-Cs complete genomes**.

| **HRV-C** | **Complete genome size (bp)** | **GC content (%)** | **Size (bp)** | | | | | | | | | | | | |
| --- | --- | --- | --- | --- | --- | --- | --- | --- | --- | --- | --- | --- | --- | --- | --- |
|  |  |  | **5’ NCR** | **VP4** | **VP2** | **VP3** | **VP1** | **2A** | **2B** | **2C** | **3A** | **3B** | **3C** | **3D** | **3’ NCR** |
| 1515-MY-10 | 7087 | 43.1 | 611 | 201 | 786 | 699 | 825 | 426 | 291 | 975 | 228 | 66 | 549 | 1380 | 50 |
| 1570-MY-10 | 7123 | 42.3 | 620 | 201 | 795 | 711 | 828 | 426 | 297 | 978 | 225 | 66 | 549 | 1380 | 47 |
| 3430-MY-10 | 7090 | 42.7 | 611 | 201 | 789 | 699 | 816 | 426 | 291 | 975 | 228 | 66 | 549 | 1380 | 59 |
| 3805-MY-10 | 7125 | 43.0 | 617 | 201 | 795 | 711 | 837 | 426 | 297 | 978 | 225 | 66 | 549 | 1380 | 43 |
| 7383-MY-10 | 7073 | 43.8 | 614 | 201 | 783 | 702 | 816 | 426 | 291 | 975 | 228 | 66 | 549 | 1380 | 42 |
| 8097-MY-11 | 7127 | 43.0 | 640 | 201 | 786 | 708 | 822 | 426 | 297 | 975 | 225 | 66 | 549 | 1380 | 52 |
| 8713-MY-10 | 7112 | 42.6 | 615 | 201 | 801 | 705 | 828 | 426 | 297 | 978 | 225 | 66 | 549 | 1380 | 41 |
| KF958310/6331/C2 | 7087 | 43.2 | 610 | 201 | 786 | 711 | 813 | 426 | 294 | 981 | 225 | 66 | 549 | 1380 | 45 |
| EF186077/QPM/C3 | 7092 | 43.3 | 612 | 201 | 789 | 699 | 825 | 426 | 291 | 975 | 228 | 66 | 549 | 1380 | 51 |
| EF582385/024/C4 | 7099 | 42.8 | 615 | 201 | 783 | 705 | 822 | 426 | 297 | 978 | 225 | 66 | 549 | 1380 | 52 |
| EF582386/025/C5 | 7114 | 41.8 | 616 | 201 | 792 | 717 | 825 | 426 | 297 | 978 | 225 | 66 | 549 | 1380 | 42 |
| EF582387/026/C6 | 7086 | 43.0 | 611 | 201 | 786 | 699 | 825 | 426 | 291 | 975 | 228 | 66 | 549 | 1380 | 49 |
| JF317016/LZ651/C6 | 7087 | 43.1 | 611 | 201 | 786 | 699 | 825 | 426 | 291 | 975 | 228 | 66 | 549 | 1380 | 50 |
| DQ875932/NY-074/C7 | 7072 | 43.6 | 608 | 201 | 786 | 699 | 816 | 426 | 291 | 975 | 228 | 66 | 549 | 1380 | 47 |
| GQ223227/N4/C8 | 7107 | 42.6 | 619 | 201 | 786 | 708 | 828 | 426 | 297 | 978 | 225 | 66 | 549 | 1380 | 44 |
| GQ223228/N10/C9 | 7111 | 43.0 | 639 | 201 | 786 | 705 | 816 | 426 | 297 | 978 | 225 | 66 | 549 | 1380 | 43 |
| EU840952/CL170085/C11 | 7108 | 41.5 | 618 | 201 | 792 | 714 | 819 | 426 | 297 | 978 | 225 | 66 | 549 | 1380 | 43 |
| JF317017/LZY101/C12 | 7124 | 42.8 | 616 | 201 | 795 | 714 | 834 | 426 | 297 | 978 | 225 | 66 | 549 | 1380 | 43 |
| GU219984/W10/C15 | 7111 | 43.2 | 607 | 201 | 795 | 705 | 837 | 426 | 297 | 978 | 225 | 66 | 549 | 1380 | 45 |
| JF317014/LZY79/C15 | 7110 | 42.8 | 608 | 201 | 795 | 705 | 837 | 426 | 297 | 978 | 225 | 66 | 549 | 1380 | 43 |
| JF317013/LZ269/C25 | 7096 | 43.9 | 610 | 201 | 795 | 705 | 825 | 426 | 297 | 978 | 225 | 66 | 549 | 1380 | 39 |
| JN205461/WA823M02/C39 | 7099 | 42.4 | 617 | 201 | 789 | 699 | 819 | 426 | 294 | 975 | 228 | 66 | 549 | 1380 | 56 |
| KF958311/2536/C41 | 7115 | 42.8 | 625 | 201 | 795 | 705 | 822 | 426 | 297 | 978 | 225 | 66 | 549 | 1380 | 46 |
| JF317015/LZ508/C51 | 7126 | 44.0 | 641 | 201 | 786 | 708 | 813 | 426 | 297 | 975 | 225 | 66 | 549 | 1380 | 59 |
| JX291115/JAL-1/C51 | 7133 | 44.0 | 645 | 201 | 786 | 708 | 813 | 426 | 297 | 978 | 225 | 66 | 549 | 1380 | 59 |

NCR, non-coding region
