# Supplementary material for: Comparative Genetic Analyses of Human Rhinovirus C (HRV-C) Complete Genome from Malaysia
Source: Front Microbiol. 2016 Apr 29;7:543. doi: 10.3389/fmicb.2016.00543 (PMC4851184; doi:10.3389/fmicb.2016.00543)
Supplement: Supplementary file 3 [file Table3.DOC]

**Supplementary Table 3: Pairwise similarity of VP4/VP2, VP1 and complete genome nucleotide sequences of Malaysian isolates and their respective closest HRV-C references**

| **Sequence** | **Pairwise similarity (%)** | | | | | | |
| --- | --- | --- | --- | --- | --- | --- | --- |
|  | **1515-MY-10** | **1570-MY-10** | **8713-MY-10** | **3430-MY-10** | **7383-MY-10** | **3805-MY-10** | **8097-MY-11** |
|  | **026 (C6)** | **C42** | **C23** | **C22** | **pat16** | **LZY101 (C12)** | **C26** |
| **VP4/VP2 sequence** | 97 | 92 | 92 | 96 | 93 | 95 | 94 |
| **VP1 sequence** | 97 | 93 | 90 | 94 | NA | 91 | 93 |
| **Complete genome** | 96 | NA | NA | NA | NA | 92 | NA |

026 (accession number for VP4/VP2, VP1 sequences and complete genome: EF582387); C42 (accession number for VP4/VP2 and VP1 sequences: JQ994500); C23 (accession number for VP4/VP2 sequence: EU752424; accession number for VP1 sequence: HM236901); C22 (accession number for VP4/VP2 and VP1 sequences: JN621242); pat16 (accession number for VP4/VP2 sequence: EU752358); LZY101 (accession number for VP4/VP2, VP1 sequences and complete genome: JF317017); C26 (accession number for VP4/VP2 and VP1 sequences: JX193796) ; NA: not available.
